# Impact of Adjunctive Intravenous Iron Therapy on Hemoglobin Recovery in Obstetric Patients: A Retrospective Cohort Study

**DOI:** 10.3390/jcm15103766

**Published:** 2026-05-14

**Authors:** Mihaela Ichim, Mihaela Bot, Andreea Borislavschi, Alexandru Filipescu

**Affiliations:** 1 Department of Obstetrics and Gynecology, “Carol Davila” University of Medicine and Pharmacy, 8 Eroii Sanitari Blvd., 050474 Bucharest, Romania; mihaela.ichim@drd.umfcd.ro (M.I.); andreea.borislavschi@drd.umfcd.ro (A.B.); alexandru.filipescu@umfcd.ro (A.F.); 2 Department of Obstetrics and Gynecology, Elias University Emergency Hospital, 17 Mărăști Blvd., 050474 Bucharest, Romania

**Keywords:** iron therapy, hemoglobin recovery, obstetric patients

## Abstract

**Background**: Postpartum anemia frequently requires packed red blood cell (PRBC) transfusion; however, the contribution of intravenous iron to early hematologic recovery remains uncertain. This study evaluated hemoglobin dynamics following transfusion and the effect of adjunctive intravenous iron therapy. **Methods**: In this retrospective cohort study, 61 obstetric patients requiring PRBC transfusion were included. Hematologic parameters were assessed at admission, postoperatively, post-transfusion, and at day 7. Patients were stratified according to the administration of intravenous ferric carboxymaltose. Hemoglobin changes and reticulocyte response were analyzed, including a subgroup receiving two PRBC units. **Results**: Mean hemoglobin decreased from 9.36 ± 1.16 g/dL at admission to 6.33 ± 1.05 g/dL postoperatively, increased to 8.32 ± 0.78 g/dL after transfusion, and reached 8.75 ± 0.97 g/dL at day 7 (*p* < 0.001). No significant differences between groups were observed before or immediately after transfusion. At day 7, hemoglobin was significantly higher in patients receiving intravenous iron compared with those without supplementation (9.63 ± 1.03 vs. 8.03 ± 0.36 g/dL, *p* < 0.001). The increase in hemoglobin from post-transfusion to day 7 was greater in the iron group (1.14 ± 0.84 vs. −0.15 ± 0.33 g/dL, *p* < 0.001), accompanied by a higher reticulocyte response (2.56 ± 0.49% vs. 0.93 ± 0.26%, *p* < 0.001). Similar findings were observed in the two-unit subgroup. **Conclusions**: Adjunctive intravenous iron was associated with improved early hemoglobin recovery after transfusion without affecting immediate correction, supporting its clinical use in obstetric patients.

## 1. Introduction

Postpartum anemia remains a common clinical challenge, affecting a substantial proportion of obstetric patients worldwide and contributing to maternal morbidity, delayed recovery, and impaired quality of life. It is most frequently the result of peripartum blood loss superimposed on pre-existing iron deficiency or anemia during pregnancy. According to the World Health Organization (WHO), anemia affects approximately 36% of pregnant women globally, with iron deficiency representing the leading cause [1].

In the setting of moderate to severe anemia, packed red blood cell (PRBC) transfusion is frequently required to achieve rapid correction of hemoglobin levels and restore adequate tissue oxygenation. While transfusion provides an immediate increase in circulating hemoglobin, its effect is limited to short-term correction and does not address the underlying iron deficit. Moreover, transfusion carries potential risks, including immunologic reactions and transfusion-related complications, underscoring the importance of optimizing alternative or adjunctive therapeutic strategies [2,3].

Several studies have confirmed that intravenous iron leads to a more rapid and sustained increase in hemoglobin levels compared to oral iron, particularly in the postpartum setting. In addition to improving hemoglobin, intravenous iron has been associated with faster replenishment of iron stores and improved clinical recovery [4,5].

Intravenous iron therapy has gained increasing attention as an effective treatment for iron deficiency anemia in the peripartum period. Ferric carboxymaltose allows the administration of high-dose iron in a single infusion and has demonstrated efficacy in improving hemoglobin levels and replenishing iron stores in postpartum patients. Compared to oral iron supplementation, intravenous formulations are associated with faster hematologic recovery and improved tolerability [4].

Intravenous ferric carboxymaltose has also been shown to be safe and well-tolerated in postpartum patients, with a low incidence of adverse events and a favorable hematologic response. In a clinical study evaluating its use in postpartum anemia, ferric carboxymaltose resulted in significant increases in hemoglobin levels within a short timeframe. Similarly, in our cohort, intravenous iron therapy was well tolerated, with no adverse events reported, and was associated with improved hemoglobin recovery at day 7 [4].

Despite these advantages, the role of intravenous iron in patients who have already received PRBC transfusions remains insufficiently defined. While the immediate hematologic response to transfusion is well established, its effect is transient, and the contribution of intravenous iron to subsequent hemoglobin recovery has not been fully elucidated. In particular, it remains unclear whether intravenous iron can enhance post-transfusion hematologic recovery and support sustained erythropoiesis in the early postpartum period [6].

Therefore, this study aimed to evaluate the dynamic changes in hemoglobin levels in transfused obstetric patients and to assess the impact of adjunctive intravenous iron therapy on early hematologic recovery. In addition, we sought to explore the relationship between transfusion exposure and iron supplementation by analyzing hemoglobin evolution and reticulocyte response, including in a subgroup of patients receiving comparable transfusion volumes.

## 2. Materials and Methods

The study was designed as a retrospective observational cohort analysis conducted at Elias University Emergency Hospital, including obstetric patients (pregnant or postpartum) requiring PRBC transfusion during the study period (September 2022–December 2025) who met the eligibility criteria (Table 1).

Clinical and demographic data were extracted from medical records, including age, comorbidities (arterial hypertension and gestational diabetes/diabetes mellitus), obstetric characteristics, and mode of delivery. Information regarding perioperative management and PRBC transfusion, including the number of PRBC units administered and use of intravenous iron therapy (ferric carboxymaltose 1000 mg), administered at least 24 h after transfusion (in the recovery phase, when erythropoiesis becomes the primary mechanism of hemoglobin restoration), was also collected.

Anemia in the obstetric setting is commonly defined as a hemoglobin level below 11 g/dL during pregnancy and below 10 g/dL in the postpartum period [7]. The decision to initiate PRBC transfusion is guided not only by hemoglobin levels (generally below 7 g/dL) but also by clinical status, including hemodynamic stability, ongoing bleeding, and the presence of symptoms. In stable patients, restrictive transfusion strategies are generally recommended, with transfusion reserved for more severe anemia or symptomatic cases.

Ferric carboxymaltose is administered as a high-dose intravenous infusion. In our cohort, a dose of 1000 mg was administered over a short period of time.

Hematological parameters were evaluated at four timepoints: at admission, postoperatively, after transfusion, and on post-transfusion day 7. Complete blood count parameters were recorded at each time point, including hemoglobin levels, red blood cell indices, and platelet count. Reticulocyte count was assessed on post-transfusion day 7.

The primary objective of the study was to evaluate changes in hemoglobin levels across the four time points. Secondary objectives included the assessment of dynamic changes in erythrocyte indices and platelet count, as well as the evaluation of early hematological recovery.

Statistical analysis was performed using standard statistical methods. Continuous variables were expressed as mean ± standard deviation (SD), and categorical variables as frequencies and percentages. Comparisons between groups were performed using Student’s t-test, while categorical variables were analyzed using the chi-square or Fisher’s exact test, as appropriate. Changes in hemoglobin levels between timepoints were evaluated, and differences between groups were assessed accordingly. A repeated-measures analysis of variance (ANOVA) was performed to evaluate changes in hemoglobin levels over time and to assess the interaction between time and intravenous iron therapy. A multivariable linear regression analysis was performed to evaluate the independent association between intravenous iron therapy and hemoglobin levels at day 7. Postoperative hemoglobin was selected as the marker of anemia severity, as it reflects the clinical baseline at which transfusion and intravenous iron administration are typically decided in routine practice. A sensitivity analysis was performed by additionally including admission hemoglobin in the regression model to assess the robustness of the findings.

A *p*-value < 0.05 was considered statistically significant.

The study was conducted in accordance with the principles of the Declaration of Helsinki. Ethical approval was obtained from the local ethics committee (7011/18 August 2022). Due to the retrospective design of the study, informed consent was waived. Patient data were anonymized before analysis to ensure confidentiality.

## 3. Results

The study cohort included 61 obstetric patients. The mean age of the population was 30.9 ± 8.7 years, with values ranging from 20 to 51 years. When stratified according to intravenous iron therapy, 34 patients did not receive iron supplementation, while 27 patients were treated with ferric carboxymaltose. The mean age was 31.3 ± 9.5 years in the non-iron group and 30.3 ± 7.6 years in the iron-treated group.

Body mass index (BMI) was available for all patients. The mean BMI of the overall cohort was 29.4 ± 4.6 kg/m^2^. When stratified by intravenous iron therapy, the mean BMI was 29.7 ± 4.7 kg/m^2^ in patients who did not receive iron supplementation and 29.0 ± 4.5 kg/m^2^ in those treated with ferric carboxymaltose.

The mean length of hospital stay was 6.8 ± 4.9 days, with slightly higher values observed in patients receiving intravenous iron therapy compared to those without iron supplementation (7.4 ± 6.6 vs. 6.3 ± 2.7 days).

Associated comorbidities were present in 39.3% of patients, including arterial hypertension (21.3%), diabetes mellitus or gestational diabetes (8.2%), and minor hematological conditions not meeting exclusion criteria (9.8%). Allergies were reported in 19.7% of cases, while 27.9% of patients were active smokers.

Regarding obstetric characteristics, the majority of patients underwent cesarean delivery (68.9%), while 23.0% had vaginal delivery. A small proportion required cesarean hysterectomy (6.6%), and one patient (1.6%) was undelivered at the time of evaluation.

In terms of obstetric history at admission, 29.5% of patients were primiparous, and 24.6% were multiparous. A history of uterine scarring was present in 44.3% of cases, including patients with previous cesarean section or myomectomy, while 1.6% of patients were in the postpartum period.

Chronic anemia was present in 50.8% of patients, while 75.4% had acute anemia at the time of admission.

At admission, 34.4% of patients were receiving medical treatment, most commonly methyldopa (11.5%) or combined iron and methyldopa therapy (18.0%). Iron supplementation alone was reported in 4.9% of cases, while no patients were receiving anticoagulant or antiplatelet therapy.

At admission, the mean hemoglobin level in the overall cohort was 9.36 ± 1.16 g/dL. When stratified by intravenous iron therapy, patients who received ferric carboxymaltose had lower baseline hemoglobin levels compared to those without iron supplementation (9.01 ± 1.46 vs. 9.64 ± 0.78 g/dL), although this difference did not reach statistical significance (*p* = 0.07). The mean hematocrit was 29.1 ± 3.5%, while the red blood cell count averaged 3.80 ± 0.42 × 10^6^/µL. Platelet count at admission was 281 ± 78 × 10^3^/µL.

At admission, 23.0% of patients were receiving iron supplementation (alone or in combination with methyldopa). Baseline hemoglobin levels were similar between patients with and without prior iron therapy (9.35 ± 0.77 vs. 9.36 ± 1.27 g/dL, *p* = 0.98). When stratified by intravenous iron administration, no significant differences in baseline hemoglobin were observed in the non-iron-treated group (*p* = 0.41). In contrast, among patients who received ferric carboxymaltose, baseline hemoglobin values were comparable between those with and without prior iron supplementation (*p* = 0.52).

**Figure 1:**
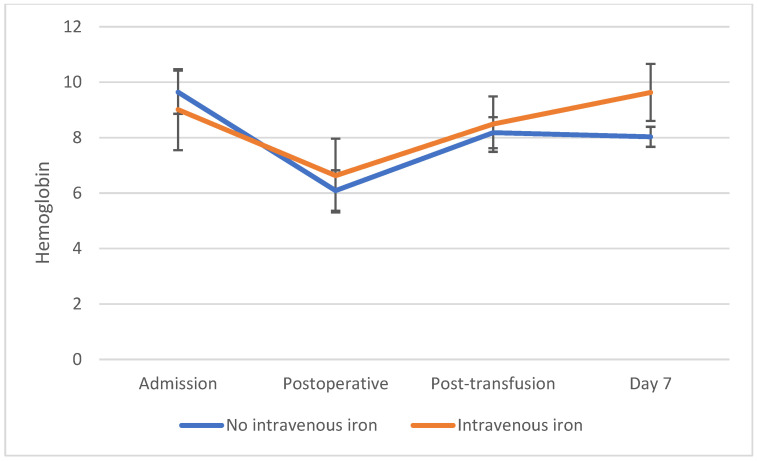
Hemoglobin evolution.

Hemoglobin levels showed significant dynamic changes across the four evaluated timepoints (*p* < 0.001). In the overall cohort, mean hemoglobin decreased from 9.36 ± 1.16 g/dL at admission to 6.33 ± 1.05 g/dL postoperatively. Following transfusion, hemoglobin increased to 8.32 ± 0.78 g/dL, with a further rise to 8.75 ± 0.97 g/dL at post-transfusion day 7 (Table 2, Figure 1).

To account for repeated measurements over time, a repeated-measures analysis was performed. A significant interaction between time and intravenous iron therapy was observed (*p* < 0.001), indicating that hemoglobin evolution over time differed between patients with and without intravenous iron supplementation.

When stratified by intravenous iron therapy, no statistically significant differences were observed between groups at admission (*p* = 0.07), postoperatively (*p* = 0.08), or immediately after transfusion (*p* = 0.15). However, hemoglobin levels at day 7 were significantly higher in patients treated with intravenous iron compared to those without iron supplementation (9.63 ± 1.03 vs. 8.03 ± 0.36 g/dL, *p* < 0.001). 

The mean decrease in hemoglobin from admission to the postoperative period was 3.03 g/dL. PRBC transfusion resulted in an average increase of 1.99 g/dL, followed by an additional increase of 0.43 g/dL between the post-transfusion measurement and day 7. The decrease in hemoglobin from admission to the postoperative period was significantly greater in patients who did not receive intravenous iron compared to those treated with ferric carboxymaltose (−3.55 ± 0.88 vs. −2.38 ± 1.37 g/dL, *p* = 0.002) (Table 3).

The increase in hemoglobin following transfusion was comparable between groups (*p* = 0.28). However, from post-transfusion to day 7, a significant difference was observed, with patients receiving intravenous iron showing a marked increase in hemoglobin levels, whereas those without iron supplementation showed no further improvement (1.14 ± 0.84 vs. −0.15 ± 0.33 g/dL, *p* < 0.001) (Table 4).

The mean number of transfused PRBC units was 2.2 ± 1.3 per patient (Table 4).

**Figure 2:**
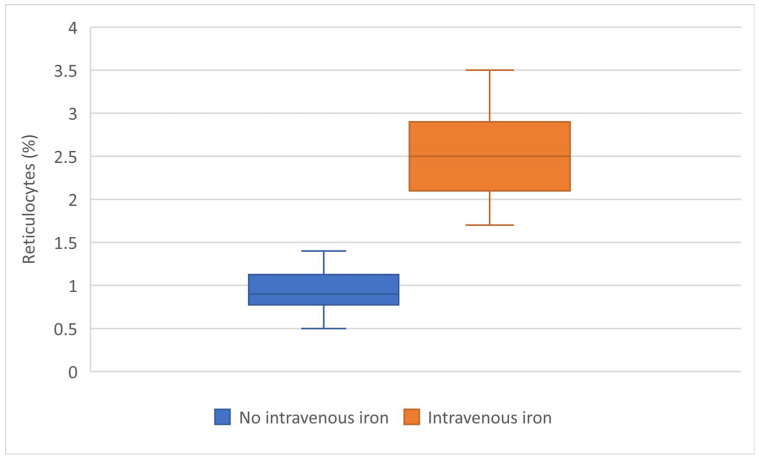
Reticulocyte levels at post-transfusion day 7 according to intravenous iron therapy. Boxplots represent the distribution of reticulocyte percentages, including median, interquartile range, and minimum–maximum values, in patients with and without intravenous iron supplementation.

To reduce the influence of transfusion volume, a subgroup analysis was performed in patients receiving two units of PRBC. Post-transfusion hemoglobin levels were comparable between patients with and without intravenous iron therapy (8.10 ± 0.45 vs. 8.90 ± 1.10 g/dL, *p* = 0.06). However, at post-transfusion day 7, patients treated with intravenous iron demonstrated significantly higher hemoglobin levels compared to those who did not receive iron supplementation (9.63 ± 1.03 vs. 8.03 ± 0.36 g/dL, *p* < 0.001). The change in hemoglobin from post-transfusion to day 7 further highlighted this difference, with a marked increase observed in the intravenous iron group, whereas hemoglobin levels remained stable or slightly decreased in the non-iron-treated group (1.14 ± 0.84 vs. −0.15 ± 0.33 g/dL, *p* < 0.001). This was associated with a significantly higher reticulocyte response in patients receiving intravenous iron (2.56 ± 0.49% vs. 0.93 ± 0.26%, *p* < 0.001), suggesting enhanced erythropoietic activity (Table 5, Figure 2).

No transfusion-related adverse events were observed during the study period. Intravenous iron therapy was also well tolerated, with no documented adverse reactions across the entire cohort.

A multivariable linear regression analysis was performed to evaluate factors associated with hemoglobin levels at day 7. After adjustment for postoperative hemoglobin and the number of transfused PRBC units, intravenous iron therapy remained independently associated with higher hemoglobin levels at day 7 (B = 0.84, 95% CI: 0.44–1.24, *p* < 0.001). Postoperative hemoglobin was also independently associated with day-7 hemoglobin levels (B = 0.42, 95% CI: 0.23–0.60, *p* < 0.001). The number of transfused PRBC units was not significantly associated with day-7 hemoglobin after adjustment (B = 0.12, 95% CI: −0.02–0.26, *p* = 0.098). The model explained 53.3% of the variability in day-7 hemoglobin levels (R^2^ = 0.533) (Table 6).

In a sensitivity analysis additionally including admission hemoglobin, intravenous iron therapy remained significantly associated with higher hemoglobin levels at day 7, confirming the robustness of the main findings.

## 4. Discussion

In this study, intravenous iron therapy was associated with a different pattern of hemoglobin recovery compared to transfusion alone.

At baseline, patients who received intravenous iron tended to have lower hemoglobin levels, suggesting that iron was preferentially administered in more anemic patients. However, despite this initial difference, hemoglobin values became significantly higher at post-transfusion day 7 in the iron-treated group.

The early increase in hemoglobin was primarily driven by PRBC transfusion, as shown by the comparable post-transfusion hemoglobin levels between groups, including in the subgroup receiving two units of PRBC. In contrast, the subsequent evolution differed significantly: patients treated with intravenous iron showed a sustained increase in hemoglobin, whereas those without iron supplementation had stable or slightly decreasing values.

This divergence was confirmed by the analysis of hemoglobin changes between timepoints. The decrease from admission to postoperative values was greater in patients without iron therapy, while the increase from post-transfusion to day 7 was significantly higher in the iron-treated group.

The subgroup analysis of patients receiving two units of PRBC further clarified this relationship. By partially controlling for transfusion exposure, it showed that the additional increase in hemoglobin at day 7 was associated with intravenous iron rather than to differences in transfusion.

Although the non-randomized design may introduce confounding, the subgroup analysis of patients receiving two units of PRBC allowed partial control of transfusion exposure. The persistence of significant differences in hemoglobin recovery within this subgroup strengthens the association between intravenous iron therapy and improved post-transfusion hematologic recovery.

This pattern was paralleled by a significantly higher reticulocyte response in the iron-treated group, indicating increased erythropoietic activity. Together, these findings suggest that intravenous iron does not contribute to immediate hemoglobin correction but plays a key role in post-transfusion hematologic recovery.

From a clinical perspective, these results support a complementary role of intravenous iron alongside transfusion, particularly in patients with moderate anemia, where it may enhance recovery without increasing transfusion requirements.

Both transfusion and intravenous iron therapy were well tolerated, with no documented adverse events identified in the study cohort.

Previous evidence has demonstrated that intravenous ferric carboxymaltose is more effective than oral iron in the management of postpartum iron deficiency anemia, resulting in a more rapid increase in hemoglobin levels and improved replenishment of iron stores. In a study, women treated with ferric carboxymaltose achieved significantly higher hemoglobin levels over a shorter period compared to those receiving oral iron therapy [8]. These findings are consistent with our results, where patients receiving intravenous iron showed a significantly greater increase in hemoglobin at day 7, despite comparable post-transfusion values, suggesting that intravenous iron appears to be associated primarily with sustained hematologic recovery rather than immediate correction.

The efficacy and safety of intravenous ferric carboxymaltose in postpartum anemia have been further supported by another study, which demonstrated that this formulation allows rapid correction of hemoglobin levels and effective replenishment of iron stores with a favorable safety profile. The authors also highlighted the practical advantages of intravenous administration, including reduced need for prolonged treatment and improved patient compliance [9]. These findings are in line with our results, where intravenous iron was well tolerated and associated with a more pronounced increase in hemoglobin at day 7, reinforcing its role in facilitating early hematologic recovery in the postpartum period.

In the peripartum setting, another study demonstrated that intravenous ferric carboxymaltose is an effective and safe option for the treatment of iron deficiency anemia during pregnancy, contributing to improved hemoglobin levels before delivery. Their findings support the concept that early correction of iron deficiency can optimize hematologic status in obstetric patients [10]. In our study, although iron was administered after transfusion, a similar beneficial effect was observed in the subsequent phase, with intravenous iron is associated with sustained increase in hemoglobin levels, suggesting that adequate iron availability remains essential for effective erythropoietic recovery.

More recent evidence has confirmed the efficacy of intravenous ferric carboxymaltose in the management of iron deficiency anemia during pregnancy, demonstrating superior hematologic outcomes compared to oral iron therapy. In a study, intravenous iron resulted in a faster and more pronounced increase in hemoglobin levels, supporting its use in cases requiring rapid correction [11]. Although our study focused on the post-transfusion period, these findings are consistent with our observation that intravenous iron may be associated with enhanced hemoglobin recovery, particularly in the days following transfusion, when endogenous erythropoiesis becomes the primary mechanism of correction.

Intravenous iron is highly effective in replenishing iron stores and increasing hemoglobin levels in iron deficiency anemia, with a rapid hematologic response observed within days and maximal improvement achieved within approximately three weeks [7,12,13]. The adoption of restrictive transfusion thresholds has resulted in more patients remaining anemic after the acute phase, emphasizing the importance of additional strategies, such as iron therapy, to support subsequent hemoglobin recovery [7,14,15].

While the beneficial effects of intravenous iron in the treatment of iron deficiency anemia are well established, most previous studies have focused on its role as an alternative to transfusion or as a primary treatment in postpartum anemia [4,8,9,10,11]. In contrast, the present study specifically evaluates the association between intravenous iron therapy and early hematologic recovery following PRBC transfusion, a clinical scenario that has been less extensively explored. Our findings suggest that intravenous iron may be associated with enhanced hemoglobin recovery and increased reticulocyte response in the early post-transfusion period, supporting its potential complementary role alongside transfusion rather than as a replacement strategy. This distinction is clinically relevant, as it reflects real-world management in patients requiring immediate correction of anemia, followed by optimization of subsequent erythropoietic recovery.

In this context, our findings further support the potential complementary role of intravenous iron following PRBC transfusion in obstetric patients. While intravenous iron is increasingly used as part of patient blood management strategies to reduce or avoid the need for PRBC transfusion in iron deficiency anemia, its role as an adjunctive therapy following transfusion remains insufficiently defined [10]. Our findings suggest that intravenous iron does not influence immediate hemoglobin correction but significantly enhances recovery in the early post-transfusion period, possibly reflecting increased erythropoietic activity, as reflected by increased reticulocyte response.

In critically ill patients, transfusion has been shown to provide only a limited and transient increase in hemoglobin levels over time. Chant et al. reported that hemoglobin values remained relatively stable after the initial post-transfusion correction, with no significant progressive increase observed during follow-up. These findings support the concept that transfusion alone does not lead to sustained hematologic improvement, which is consistent with our results, where hemoglobin levels remained stable or slightly decreased in patients who did not receive intravenous iron [16]. Postoperative hemoglobin recovery has been shown to occur gradually over time and is largely dependent on endogenous erythropoiesis rather than transfusion alone. Wallis et al. demonstrated that significant hemoglobin improvement occurs beyond the first postoperative week, supporting the concept that later recovery reflects physiological red blood cell production, in line with our findings [17].

Laine et al. demonstrated that hemoglobin levels at 7 days did not significantly differ between patients receiving different transfusion strategies, despite variations in transfusion volume. This suggests that transfusion alone does not determine later hemoglobin levels, supporting our findings that additional factors, such as intravenous iron, may play a key role in post-transfusion recovery [18].

Changes in hemoglobin following transfusion may not be sustained over time, as shown by studies evaluating hemoglobin levels at later timepoints. In this context, hemoglobin variation at 7 days reflects not only the immediate effect of transfusion but also ongoing physiological processes, including erythropoiesis and underlying clinical factors [19]. This supports our findings, where differences in hemoglobin at day 7 were more pronounced in patients receiving intravenous iron.

In the adjusted analysis, intravenous iron therapy remained significantly associated with higher hemoglobin levels at day 7, independent of postoperative hemoglobin and transfusion volume. Postoperative hemoglobin was selected as the marker of anemia severity because it represented the clinical baseline at which transfusion and intravenous iron administration were considered. These findings suggest that the association between intravenous iron therapy and early hemoglobin recovery was not solely explained by postoperative anemia severity or the number of transfused PRBC units. However, because of the retrospective and non-randomized design, residual confounding and confounding by indication cannot be excluded; therefore, these results should be interpreted as associations rather than causal effects.

The robustness of the findings was further supported by sensitivity analysis, in which the association between intravenous iron therapy and improved hemoglobin recovery remained significant after additional adjustment for admission hemoglobin. Although more advanced approaches such as mixed-effects models or propensity score methods could be considered, the present analysis included the main clinically relevant confounders and was supported by sensitivity analysis, providing consistent results.

Intravenous iron has been increasingly integrated into patient blood management strategies, particularly in perioperative settings, where it has been shown to reduce transfusion requirements and support hematologic recovery. However, its role is primarily complementary, as it does not replace transfusion in cases requiring rapid correction. These observations are consistent with our findings, suggesting that intravenous iron may enhance post-transfusion recovery rather than contribute to immediate hemoglobin correction [20,21,22].

The main limitations of this study include its retrospective design and the relatively small sample size, which may limit the generalizability of the findings. In addition, the lack of randomization may introduce selection bias, as intravenous iron therapy was more frequently administered in patients with more severe anemia, raising the possibility of confounding by indication. Although subgroup analysis and adjusted analyses were performed to partially control for transfusion exposure and anemia severity, residual confounding factors cannot be excluded. Furthermore, iron status markers, such as ferritin, transferrin saturation, and serum iron, were not consistently available, as these parameters were not routinely assessed in all patients due to the retrospective nature of the study. As a result, baseline iron deficiency could not be systematically confirmed, and the mechanistic interpretation of the response to intravenous iron remains limited. In addition, although a general patient blood management strategy has been implemented at the institutional level, a standardized protocol specifically tailored to obstetrics and gynecology is still under development; therefore, the decision to administer intravenous iron remained based on clinician judgment, which may have introduced variability in treatment allocation. Finally, the short follow-up period, limited to the in-hospital setting, restricts the assessment of long-term hematologic outcomes and prevents evaluation of clinically relevant endpoints such as symptom improvement or quality of life. Therefore, the findings of this study should be interpreted as associations rather than causal relationships.

## 5. Conclusions

Intravenous iron therapy was associated with improved hemoglobin recovery following PRBC transfusion in obstetric patients. While transfusion remained the primary determinant of early hemoglobin correction, intravenous iron was associated with a sustained increase in hemoglobin levels in the days following transfusion. This effect was supported by a significantly higher reticulocyte response, suggesting enhanced erythropoietic activity. In patients receiving comparable transfusion volumes, intravenous iron was associated with superior hemoglobin levels at day 7, suggesting a potential role in optimizing post-transfusion recovery rather than immediate correction.

The present study adds to the existing literature by providing a dynamic evaluation of the hemoglobin and reticulocyte response following transfusion, highlighting the complementary role of intravenous iron in enhancing hematologic recovery during the early post-transfusion period.

However, several limitations should be acknowledged. The study design and sample size may limit the generalizability of the findings, and potential confounding factors related to patient characteristics and clinical management cannot be fully excluded. In addition, the short follow-up period restricts the assessment of long-term outcomes.

Future research should focus on larger, prospective studies to confirm these findings, evaluate optimal dosing strategies for intravenous iron, and assess long-term clinical outcomes, including recovery, quality of life, and the potential reduction in transfusion requirements.

## Figures and Tables

**Table 1:** Inclusion and exclusion criteria.

Inclusion Criteria	Exclusion Criteria
Obstetric patients (pregnant > 32 weeks of gestation or postpartum) requiring PRBC transfusion Patients with available hematological data at predefined timepoints	Incomplete laboratory data Severe or clinically significant hematological disorders (e.g., hemoglobinopathies or bone marrow disorders) that could directly affect erythropoiesis and hemoglobin dynamics

**Table 2:** Hemoglobin evolution and *p*-values.

Timepoint	Total Mean	Total SD	No Iron Mean	No Iron SD	With Iron Mean	With Iron SD	*p* Value
Admission	9.36	1.16	9.64	0.78	9.01	1.46	0.07
Postoperative	6.33	1.05	6.09	0.73	6.63	1.33	0.08
Post-transfusion	8.32	0.78	8.18	0.56	8.49	1.00	0.15
Day 7	8.75	0.97	8.03	0.36	9.63	1.03	<0.001

SD: standard deviation.

**Table 3:** Mean hemoglobin change (ΔHb, g/dL) calculated between consecutive timepoints (paired analysis).

Change	Overall Mean	Overall SD	No Iron Mean	No Iron SD	With Iron Mean	With Iron SD	*p* Value
Admission → Postop	−3.03	1.14	−3.55	0.88	−2.38	1.37	0.002
Postop → Post-transfusion	1.99	0.84	2.09	0.63	1.86	1.05	0.28
Post-transfusion → Day 7	0.43	0.86	−0.15	0.33	1.14	0.84	<0.001

**Table 4:** The mean number of transfused packed red blood cell units.

Parameter	Value
Mean units transfused	2.2
Standard deviation	1.3
Minimum	1
Maximum	10

**Table 5:** Hemoglobin evolution and reticulocyte response in patients receiving two units of packed red blood cells, stratified by intravenous iron therapy.

Parameter	No Iron Mean	No Iron SD	With Iron Mean	With Iron SD	*p* Value
Post-transfusion Hb (g/dL)	8.10	0.45	8.90	1.10	0.06
Day 7 Hb (g/dL)	8.03	0.36	9.63	1.03	<0.001
ΔHb (post-transfusion → day 7) (g/dL)	−0.15	0.33	1.14	0.84	<0.001
Reticulocytes at day 7 (%)	0.93	0.26	2.56	0.49	<0.001

**Table 6:** Multivariable linear regression analysis for hemoglobin at day 7.

Variable	B	95% CI	*p*-Value
Intravenous iron therapy	0.84	0.44–1.24	<0.001
Postoperative hemoglobin	0.42	0.23–0.60	<0.001
PRBC units transfused	0.12	−0.02–0.26	0.098

## Data Availability

The data supporting the findings of this study are not publicly available due to privacy and ethical restrictions, as they contain clinical information derived from medical records. Anonymized data may be made available from the corresponding author upon reasonable request and with approval from the local ethics committee.
